# Antitumor effects of a novel glucose-conjugated bacteriochlorin for photodynamic therapy

**DOI:** 10.1038/s41598-025-29901-7

**Published:** 2025-11-25

**Authors:** Yasunari Sasaki, Mamoru Tanaka, Yuki Kojima, Makiko Sasaki, Tomohiro Watada, Ryusei Yamasaki, Kanato Matsuura, Akihiro Nomoto, Shigenobu Yano, Tomokazu Yoshimura, Atsushi Narumi, Keiji Ozeki, Takaya Shimura, Eiji Kubota, Hiromi Kataoka

**Affiliations:** 1 Department of Gastroenterology and Metabolism, Nagoya City University Graduate School of Medical Science, Nagoya, Aichi Japan; 2 Department of Applied Chemistry, Graduate School of Engineering, Osaka Metropolitan University, Osaka, Japan; 3 Department of Chemistry and Biology, National Institute of Technology, Fukui College, Fukui, Japan; 4 KYOUSEI Science Center for Life and Nature, Nara Women’s University, Nara, Japan; 5 Department of Chemistry, Graduate School of Science, The University of Osaka, Osaka, Japan; 6 Department of Chemistry, Faculty of Science, Nara Women’s University, Nara, Japan; 7 Department of Polymer Science and Engineering, Graduate School of Science and Engineering, Yamagata University, Yamagata, Japan

**Keywords:** Photodynamic therapy, Photosensitizer, Bacteriochlorin, Glucose-conjugated, Talaporfin sodium, Reactive oxygen species, Biotechnology, Cancer, Drug discovery

## Abstract

Photodynamic therapy (PDT) is a minimally invasive cancer treatment that employs photosensitizers (PSs) activated by light to generate cytotoxic reactive oxygen species (ROS). Talaporfin sodium (TS), widely used in Japan, has shown limited efficacy in advanced tumors, highlighting the need for novel PSs. We developed a glucose-conjugated bacteriochlorin derivative, Glc-TFPB, designed to enhance tumor selectivity via the Warburg effect and to enable deep tissue penetration owing to its near-infrared absorption. In vitro, Glc-TFPB exhibited time-dependent cellular uptake, predominantly in lysosomes, and induced significant ROS generation and apoptosis upon PDT. Compared with TS, Glc-TFPB-PDT demonstrated markedly higher cytotoxicity 24 h after administration (IC50: 1.10 µM vs 13.60 µM). In vivo fluorescence analysis revealed peak tumor accumulation at 24 h after administration, consistent with optimal treatment timing. PDT with Glc-TFPB significantly suppressed tumor growth in xenografted mice, with greater efficacy at 24 h than at 2 h after administration. These findings indicate that Glc-TFPB is a promising next-generation PS with improved tumor selectivity, deeper tissue penetration, and superior photodynamic efficacy, supporting its potential for clinical translation.

**Supplementary Information:**

The online version contains supplementary material available at 10.1038/s41598-025-29901-7.

## Introduction

In developed countries, including Japan, cancer incidence is increasing due to an aging population. Invasive treatments, such as surgery and chemotherapy, may pose a considerable physical burden on elderly patients, necessitating the development of safer and more precise targeted therapeutic approaches. Recently, photodynamic therapy (PDT), a minimally invasive cancer therapy using photosensitizers (PSs) activated by specific light wavelengths, has attracted considerable attention. PDT reduces systemic toxicity by selectively delivering light to specific tumor sites with PS accumulation^1^. Tumor growth inhibition by PDT occurs via three primary mechanisms: (1) direct cytotoxicity mediated by reactive oxygen species (ROS), (2) vascular shutdown causing tumor infarction, and (3) activation of antitumor immune responses^2–6^.

Since its discovery in the early twentieth century, PDT has undergone extensive development and has been successfully applied in clinical practice. In gastrointestinal oncology, talaporfin sodium (TS), a second-generation PS, is predominantly used for PDT in Japan. Porfimer sodium, a first-generation PS, is associated with a high incidence of skin photosensitivity, requiring prolonged light avoidance for 4–6 weeks^7,8^. However, TS development substantially reduced skin photosensitivity and shortened light-avoidance period to less than two weeks^9–11^. TS-PDT exhibits considerable efficacy, achieving a high complete response rate in patients with esophageal cancer limited to the submucosal layer. However, complete response rate decreases to 57.1% when the tumor extends into the muscularis propria^12,13^. These reports underscore the need for novel PSs with improved tumor selectivity and enhanced therapeutic efficacy, particularly for advanced diseases. We previously reported a glucose-conjugated chlorin compound as a PS superior to conventional agents, exploiting the Warburg effect, whereby cancer cells exhibit enhanced glucose uptake compared to normal cells^14–18^. Similar observations have been reported for tumors outside the gastrointestinal tract^19^. Based on these reports, we developed a novel glucose-conjugated bacteriochlorin PS, 5,10,15,20-tetrakis(4-(β-D-glucopyranosylthio)-2,3,5,6-tetrafluorophenyl)-2,3-(methano(N-methyl)iminomethano)bacteriochlorin (Glc-TFPB). Bacteriochlorins absorb light at longer wavelengths than chlorins, enabling deeper tissue penetration and potentially improving the therapeutic efficacy^20,21^. Additionally, glucose conjugation is expected toenhance antitumor efficacy via the Warburg effect^22–25^. In this study, we evaluated the photodynamic efficacy of Glc-TFPB using in vitro and in vivo cancer models. This study provides proof of concept for Glc-TFPB as a next-generation PS with improved tumor selectivity and potency, which may contribute to the advancement of minimally invasive cancer therapies.

## Materials and methods

### Photosensitizers

**Fig. 1:**
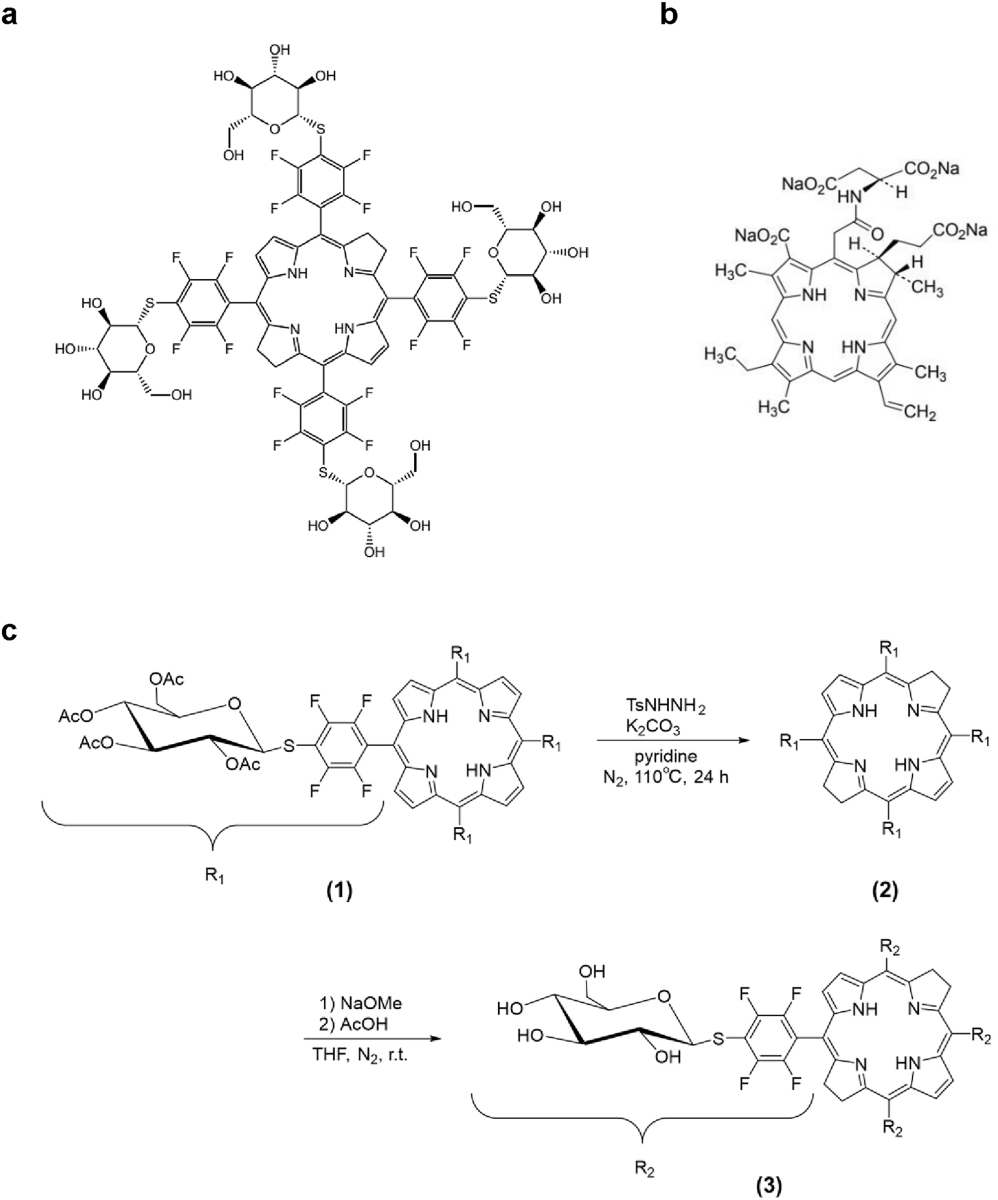
Chemical structures of photosensitizers and synthesis of 5,10,15,20-tetrakis(4-(β-D-glucopyranosylthio)-2,3,5,6-tetrafluorophenyl)-2,3-(methano(N-methyl)iminomethano)bacteriochlorin (Glc-TFPB). (**a**) Glc-TFPB: 5,10,15,20-tetrakis(4-(β-D-glucopyranosylthio)-2,3,5,6-tetrafluorophenyl)-2,3-(methano(N-methyl)iminomethano)bacteriochlorin. (**b**) TS: Mono-L-aspartyl chlorin e6. (**c**) Glucose-conjugated porphyrin (1) was converted into a bacteriochlorin derivative (2) via reduction with p-toluenesulfonyl hydrazide, and subsequent deacetylation of the glucose moiety yielded glucose-conjugated bacteriochlorin (3).

Glc-TFPB was newly synthesized and supplied by the Department of Chemistry and Biology, National Institute of Technology (Fukui College, Fukui, Japan). TS was obtained from Meiji Seika Pharma Co., Ltd. (Tokyo, Japan; Fig. 1a,b).

**Fig. 2:**
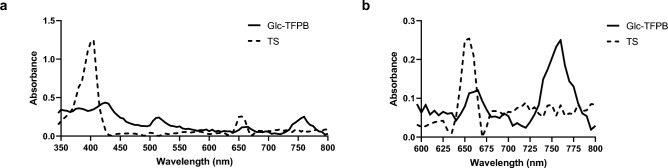
Solubility of Glc-TFPB and TS-TFPB in PBS. (**a**) Solubility of photosensitizers in PBS was assessed via ultraviolet–visible (UV–Vis) spectroscopy (350–800 nm). (**b**) Enlarged view of the absorption spectrum in the wavelength range of 600–800 nm.

Glucose-conjugated porphyrin was synthesized as previously described^26,27^. Subsequently, it was converted into a bacteriochlorin derivative according to a modified version of a previously reported method^28^. In a flask equipped with a condenser, glucose-conjugated porphyrin (202.6 mg; 0.086 mmol) was dissolved in pyridine (20 mL), and *p*-TsNHNH_2_ (598.6 mg; 3.21 mmol) and K_2_CO_3_ (708.7 mg; 5.12 mmol) were added. The mixture was refluxed under N_2_ atmosphere, and the solvent was removed under reduced pressure after 24 h. Crude product was dissolved in dichloromethane and filtered to yield the bacteriochlorin derivative, which was characterized via ultraviolet–visible (UV–Vis) spectroscopy (λ_max_: 349, 378, 418, 511, 666, and 753 nm in dichloromethane). For deacetylation of the glucose moiety, the bacteriochlorin derivative (111.1 mg; 0.047 mmol) was dissolved in tetrahydrofuran (10 mL), and NaOMe (127.3 mg; 2.36 mmol) was added. The reaction mixture was stirred for 6.5 h at an ambient temperature under N_2_ atmosphere. The resulting mixture was neutralized with AcOH (130 µL) and dialyzed against water using Spectra/Por dialysis tubing (MWCO 1000). After three days, the resulting precipitate was filtered and dried in vacuo to give glucose-conjugated bacteriochlorin as a green powder (Fig. 1c). The structure and purity of the synthesized glucose-conjugated bacteriochlorin were confirmed by NMR spectroscopy (Supplementary Fig. 1A), UV–Vis absorption spectroscopy (Supplementary Fig. 1B), and mass spectrometry (Supplementary Figs. 2 and 3). ^1^H NMR spectra were measured in CDCl_3_ with Me_4_Si as an internal standard and recorded on a JEOL JNM-ECX400 spectrometer (400 MHz; CDCl3 δ 7.26 ppm; 16 scans). UV–Vis spectroscopy was then performed in dichloromethane (λmax: 350, 380, 413, 510, 661, and 750 nm). Glucose-conjugated bacteriochlorin was dissolved in dimethyl sulfoxide (1–2 mL), without further purification, so the concentration of bacteriochlorin derivative was calculated (ε_748_ = 2.39 × 10^4^ M^-1^ cm^-1^) as a stock solution (1.5–3.5 × 10^–5^ M; Fig. 2)^29^.

### Cell culture

HCT116 human colorectal cancer cell line (lot no. 3903110; American Type Culture Collection, Manassas, VA, USA) was cultured in the Roswell Park Memorial Institute-1640 medium (Wako, Tokyo, Japan) supplemented with 10% fetal bovine serum and 1% ampicillin–streptomycin. Cells were maintained at 37 °C in a humidified atmosphere containing 5% CO_2_. All experiments were performed using cells with fewer than 20 passages after thawing.

### Animals and tumor models

Four-week-old pathogen-free female nude mice (BALB/c Slc-nu/nu; weight, 15–20 g) were purchased from Japan SLC (Shizuoka, Japan). The animals were acclimated in an animal facility for two weeks before the experiment. Xenograft tumor models were established via subcutaneous injection of 1 × 10⁶ HCT116 cells suspended in 100 μL phosphate-buffered saline (PBS). The health status of the mice was monitored throughout the study. Mice with tumor volumes of 1,000 mm^3^ were designated for euthanasia. No mice were found to be unhealthy or dead during the experimental period. Anesthesia was induced by intraperitoneal injection of ketamine (100 mg/kg) and xylazine (10 mg/kg) dissolved in physiological saline. Euthanasia was performed by carbon dioxide inhalation followed by cervical dislocation. No patient-derived materials were used in this study. Therefore, no patient-specific ethical approval was required. All animal experiments were conducted using protocols approved by the Nagoya City University Center for Experimental Animal Science, and the mice were treated according to the guidelines of the Nagoya City University for Animal Experiments (permit number 25-004). All experiments involving animals were reported in accordance with ARRIVE guidelines.

### Cellular uptake in cancer cells

HCT116 cells were seeded in a 12-well plate at a density of 5 × 10^4^ cells/well in a culture medium and incubated at 37 °C in 5% CO_2_ for 24 h. The medium was replaced with a fresh medium containing 10 µM Glc-TFPB, and the cells were incubated for 0, 1, 2, 4, 24, and 48 h to evaluate cellular uptake of the PS. After incubation, the cells were washed with PBS and detached using trypsin–EDTA. Fluorescence intensity was measured via flow cytometry using the APC-Cy7 channel (excitation: 633 nm; emission: 780/60 nm), as Glc-TFPB exhibits near-infrared fluorescence in this range. Flow cytometric analysis was conducted using FACS Canto II (BD Biosciences, Franklin Lakes, NJ, USA), and 10,000 gated events per sample were collected and analyzed using FlowJo software (BD Biosciences).

### Intracellular localization

HCT116 cells were seeded in a culture plate and incubated with 10 µM Glc-TFPB for 24 h. After incubation, lysosomes, mitochondria, the Golgi apparatus, and the endoplasmic reticulum were stained with organelle-specific fluorescent probes to assess the intracellular localization of PS. Confocal microscopy was performed using the FV3000 system (Olympus, Tokyo, Japan) and images were analyzed using FV31S-SW software (Olympus). Lysosomes were stained with 0.1 µM LysoTracker Green (Thermo Fisher Scientific, Waltham, Massachusetts, USA) at 37 °C in 5% CO₂ for 30 min. Mitochondria were labeled with 0.1 µM MitoTracker Green FM (Thermo Fisher Scientific) under the same conditions for 15 min. The Golgi apparatus was stained with 5 µM NBD C6-ceramide (Thermo Fisher Scientific) on ice at 4 °C for 30 min. The endoplasmic reticulum was stained with 1 µM ER-Tracker Green (Thermo Fisher Scientific) at 37 °C in 5% CO₂ for 30 min. Fluorescence signals were acquired using a 488-nm bandpass emission filter for organelle-specific probes and a 647-nm filter for Glc-TFPB. Fluorescence intensity profiles were obtained via confocal microscopy, and quantitative analysis was performed using cellSens imaging software (Olympus). Data are represented as mean ± standard error of the mean (SEM) of six independent experiments.

### ROS production

Intracellular ROS production was assessed using ROS detection reagents (Thermo Fisher Scientific). HCT116 cells (6 × 10^5^ cells/well) were incubated with 5 µM Glc-TFPB or TS for 24 h, followed by irradiation with a light-emitting diode (LED) (CCS Inc., Kyoto, Japan) at 735 nm for Glc-TFPB or 660 nm for TS, with an energy density of 12 J/cm^2^ and irradiance of 12 mW/cm^2^. Immediately after irradiation, the cells were detached using trypsin–EDTA and incubated with 10 μM 5-(and-6)-carboxy-2′,7′-difluorodihydrofluorescein diacetate (H₂DFFDA) for 15 min. Intracellular ROS levels were quantified via flow cytometry using FACS Canto II (excitation: 633 nm; emission: 780/60 nm).

### Singlet oxygen generation

The Singlet Oxygen Sensor Green (SOSG) reagent (Thermo Fisher Scientific) was used to detect singlet oxygen generation during PDT using Glc-TFPB. Solutions containing selected concentrations of Glc-TFPB prepared with 50 nM SOSG were irradiated with LED at 735 nm with an energy density of 12 J/cm^2^ and an irradiance of 12 mW/cm^2^. The fluorescence intensity was measured using the SpectraMax Gemini EM microplate reader (Molecular Devices, San Jose, CA, USA). The excitation wavelength was set at 490 nm, and emission spectra were recorded over range of 450–550 nm. The obtained data were analyzed using SoftMax Pro software (Molecular Devices). Representative fluorescence spectra from one of three independent experiments are shown.

### Active caspase-3 detection

HCT116 cells (3 × 105 cells/well) were incubated with 4 μM Glc-TFPB for 24 h, followed by in vitro PDT using light at 735 nm with an energy density of 12 J/cm^2^ and irradiance of 12 mW/cm^2^. After further incubation for 4 and 16 h, both the cells and culture medium were collected. Cell pellets were prepared and stained using the PE Active Caspase-3 Apoptosis Kit (BD Biosciences), according to the manufacturer’s instructions. Flow cytometric analysis was performed using FACS Canto II system, and data were analyzed using the FlowJo software.

### In vitro PDT

HCT116 cells were incubated with various Glc-TFPB or TS concentrations in a culture medium for 2 and 24 h. After incubation, the cells were washed with PBS and irradiated with LED at 735 nm for Glc-TFPB or 660 nm for TS, with an energy density of 12 J/cm2 and irradiance of 12 mW/cm^2^. Following irradiation, PBS was replaced with a fresh culture medium, and the cells were incubated overnight before analysis.

### Cell viability assay

Cell viability was assessed using the Cell Counting Kit-8 (Dojindo, Kumamoto, Japan), with cells incubated for 2 h, according to the manufacturer’s instructions. Absorbance was measured at 450 nm using a microplate spectrophotometer (SpectraMax 340PC384; Molecular Devices). Cell viability was expressed as a percentage relative to the control cells, and half-maximal inhibitory concentration (IC_50_) was determined via non-linear regression analysis.

### In vivo spectrophotometric analysis

PS accumulation in xenograft tumors was assessed using a semiconductor laser equipped with the VLD-M1 spectrometer (M&M Co., Ltd., Tokyo, Japan), which emitted laser light at a peak wavelength of 405 ± 1 nm with an output power of 140 mW. Spectral data were acquired and analyzed using dedicated software (BW-Spec V3.24; B&W TEK, Inc., Newark, DE, USA). Emission spectra showed characteristic peaks at 505 nm, corresponding to tissue autofluorescence, and 660 nm, corresponding to Glc-TFPB fluorescence. When the tumor volume reached approximately 100 mm3, Glc-TFPB was intravenously administered at a dose of 62.5 μmol/kg via the tail vein. Fluorescence intensity was measured at multiple time points (before and 0, 4, 16, 24, 48, 72, and 96 h after Glc-TFPB administration) from both the tumor surface and the adjacent normal skin tissue under the same experimental conditions. The probe tip was positioned perpendicularly to the tissue surface and kept slightly apart to ensure consistent detection geometry. To minimize variability and improve quantitative reliability, relative fluorescence intensity of PS was normalized to tissue autofluorescence. The resulting fluorescence intensity ratio (PS/autofluorescence) was used to compare Glc-TFPB accumulation in the tumor and normal skin tissues.

### In vivo PDT

When the mouse tumor volume reached approximately 100 mm3, Glc-TFPB was intravenously administered at a dose of 6.25 μmol/kg via the tail vein. Tumors were irradiated with LED at 735 nm, either 2 or 24 h after injection, at an energy density of 40 J/cm^2^ and an irradiance of 18 mW/cm2. Tumor growth was monitored every 2–3 days by measuring the tumor dimensions using Vernier calipers, and tumor volume was calculated using the following formula: (length × width × depth)/2. Statistical comparisons among groups were performed using the Holm–Sidak test.

## Results

### Glc-TFPB exhibits absorption at a longer wavelength than TS

Solubility of Glc-TFPB and TS in PBS was measured via UV–Vis spectroscopy at a concentration of 10 µM. The maximum absorption wavelength of Glc-TFPB was observed at approximately 760 nm, representing a red shift compared to that of TS (660 nm; Fig. 2).

### Glc-TFPB is slowly taken up by HCT116 cells and localized to lysosomes

**Fig. 3:**
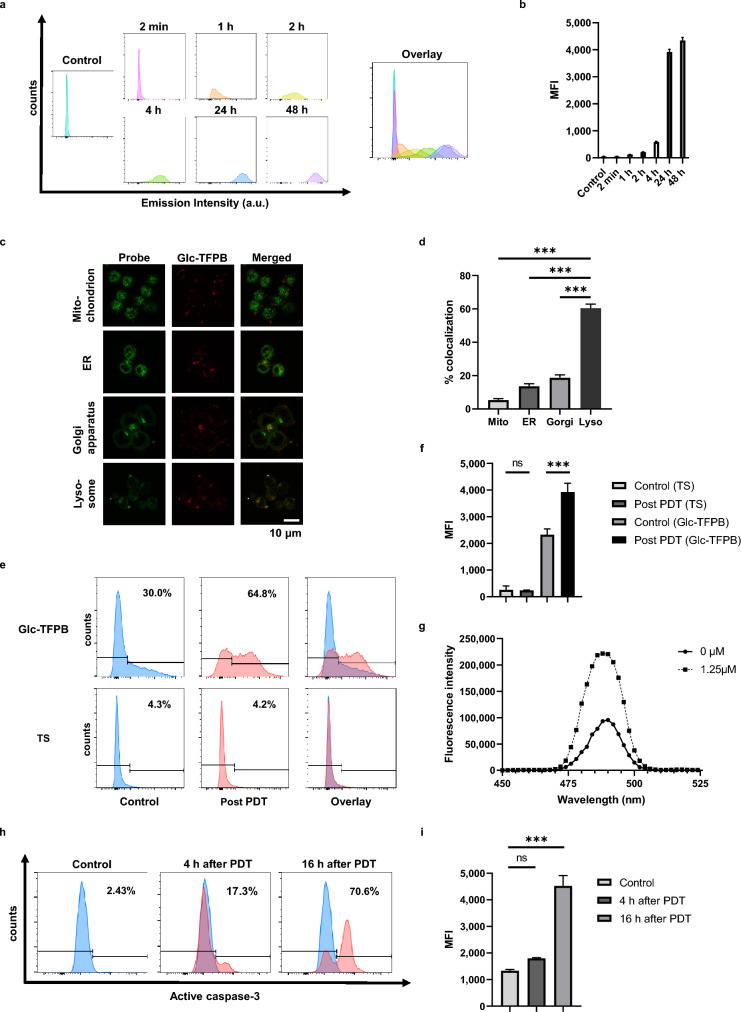
Evaluation of cellular uptake, subcellular localization, ROS and singlet oxygen generation, and apoptosis induction of photosensitizers. (**a**) Flow cytometric histograms showing time-dependent uptake of Glc-TFPB in HCT116 cells. The x-axis represents fluorescence intensity, and the y-axis represents the number of cells. (**b**) Quantification of cellular uptake expressed as mean fluorescence intensity (MFI). Data are presented as the mean ± SEM (n = 3). (**c**) Confocal microscopy images showing subcellular localization of Glc-TFPB (10 µM, 24 h) in HCT116 cells stained with organelle-specific fluorescent probes. Original magnification × 300; scale bar 10 µm. (**d**) Quantitative analysis of subcellular localization of Glc-TFPB. Data are represented as mean ± SEM (n = 6 per organelle). (**e**) Comparison of ROS generation between Glc-TFPB-PDT and TS-PDT. HCT116 cells were incubated with 5 µM Glc-TFPB or TS for 24 h, then either irradiated with light or left unirradiated, stained with H₂DFFDA, and analyzed by flow cytometry. Representative data from one of three independent experiments are shown. (**f**) Quantification of ROS generation based on MFI under different conditions (TS or Glc-TFPB treatment with or without PDT). Data are presented as mean ± SEM (n = 3). (**g**) Singlet oxygen generation detected using SOSG at selected concentrations of Glc-TFPB after light irradiation. Representative fluorescence spectra from one of three independent experiments are shown. (**h**) Flow cytometric analysis of apoptosis based on active caspase-3 levels after Glc-TFPB-PDT. HCT116 cells were incubated with 4 µM Glc-TFPB, with or without light irradiation, stained with the PE Active Caspase-3 Apoptosis Kit, and analyzed by flow cytometry. Representative histograms from one of three independent experiments are shown. (**i**) Quantification of active caspase-3 expression based on MFI. Data are presented as mean ± SEM (n = 3). Statistical analyses were performed using the Holm–Sidak test for (**d**) and Tukey’s multiple comparisons test for (**f**) and (**i**). Differences were considered statistically significant at ***p < 0.001.

Time-course analysis was performed via flow cytometry to investigate the Glc-TFPB uptake kinetics. Intracellular Glc-TFPB uptake progressively increased over the first 24 h post-administration, after which it plateaued, with no significant changes observed between 24 and 48 h (Fig. 3a,b). Subcellular localization of Glc-TFPB was determined by staining the lysosomes, mitochondria, Golgi apparatus, and endoplasmic reticulum with organelle-specific fluorescent probes. Confocal microscopy revealed that Glc-TFPB was predominantly accumulated in the lysosomes, with only minimal localization observed in the mitochondria, endoplasmic reticulum, and Golgi apparatus (***p < 0.001; Fig. 3c,d).

### Glc-TFPB-PDT induces ROS production, singlet oxygen generation, and apoptosis

ROS production and apoptosis were assessed using H₂DFFDA and the PE Active Caspase-3 Apoptosis Kit, respectively. Notably, significant ROS production was observed under PDT even at a low concentration (5 µM) of Glc-TFPB, whereas no apparent ROS production was detected in TS-PDT under the same conditions (Fig. 3e,f). To further characterize the ROS produced by Glc-TFPB-PDT, singlet oxygen generation was evaluated using SOSG, confirming that Glc-TFPB generated singlet oxygen upon light irradiation (Fig. 3g). Furthermore, the proportion of active caspase-3-positive cells was significantly higher at 16 h than at 4 h post-PDT, indicating that Glc-TFPB-PDT effectively induced apoptosis in a time-dependent manner (Fig. 3h,i).

### Glc-TFPB exhibits higher cytotoxicity than TS in vitro

**Fig. 4:**
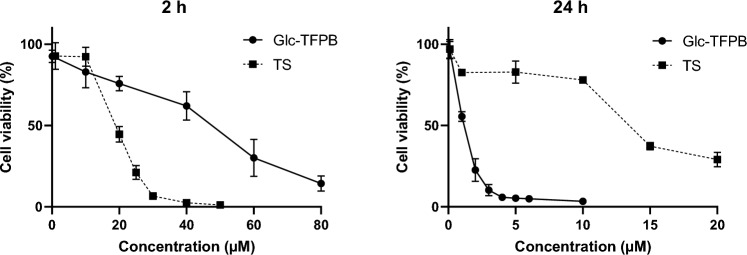
Comparison of cell viability between Glc-TFPB-PDT and TS-PDT. Cell viability was assessed using the Cell Counting Kit-8 (CCK-8) assay and expressed as the percentage relative to the control. HCT116 cells were incubated with various concentrations of TS or Glc-TFPB for 2 or 24 h, followed by irradiation with a light-emitting diode at a fluence of 12 J/cm2 (wavelengths: 735 nm for Glc-TFPB and 660 nm for TS). Data are presented as mean ± standard deviation of three independent experiments.

Cytotoxic effects of Glc-TFPB-PDT and TS-PDT on HCT116 cells were compared by evaluating their IC_50_ values. When PDT was performed 2 h after PS administration, IC_50_ value of Glc-TFPB was approximately twice that of TS, indicating its lower cytotoxic efficacy compared to that of TS-PDT (Glc-TFPB: 40.13 ± 6.22 µM; TS: 18.90 ± 0.95 µM). In contrast, when PDT was performed 24 h after administration, IC_50_ of Glc-TFPB decreased significantly, whereas that of TS remained relatively unchanged (Glc-TFPB: 1.10 ± 0.08 µM; TS: 13.60 ± 1.98 µM). Therefore, Glc-TFPB-PDT exhibited an approximately 13-fold higher cytotoxicity than TS-PDT under the tested conditions (Fig. 4 and Table 1).

**Table 1:** IC₅₀ values of TS and Glc-TFPB determined from the dose–response curves shown in Fig. 4.

	IC_50_ (μM)	
	Administration time of PS	
	2 h	24 h
TS	18.90 ± 0.95	13.60 ± 1.98
Glc-TFPB	40.13 ± 6.22	1.10 ± 0.08

### Glc-TFPB shows peak tumor accumulation 24 h after in vivo injection

**Fig. 5:**
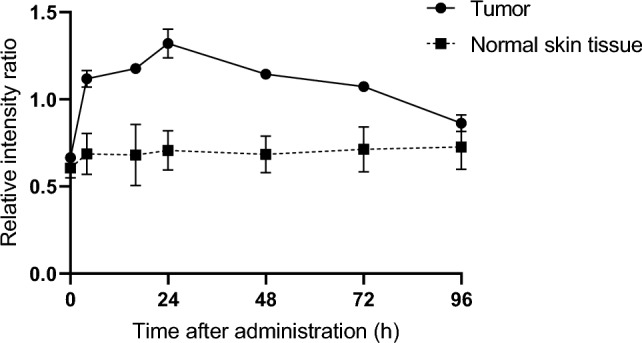
Accumulation of Glc-TFPB in mouse xenograft models. Spectral waveforms of tumor and normal tissues were recorded using VLD-M1 spectrometer. Relative fluorescence intensity of the photosensitizer and tissue autofluorescence were measured at multiple time points post-administration. Relative fluorescence intensity ratio was calculated as the ratio of photosensitizer fluorescence intensity to autofluorescence intensity in both the tumor and normal skin tissues (n = 3 per group).

Fluorescence intensities in tumors and normal tissues were measured over time using the VLD-M1 spectrometer, and Glc-TFPB accumulation in mouse xenograft models was evaluated by calculating the ratio of PS fluorescence to tissue autofluorescence. The relative fluorescence intensity ratio in normal tissues showed minimal changes after Glc-TFPB administration. In contrast, the ratio in tumor tissues peaked 24 h after administration and gradually declined thereafter, approaching levels observed in normal tissues (Fig. 5).

### Glc-TFPB-PDT significantly suppresses tumor growth in vivo

**Fig. 6:**
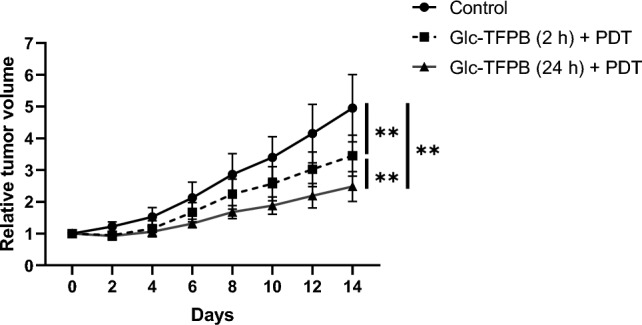
Tumor growth inhibition by PDT using Glc-TFPB in mouse xenograft models. Mice were irradiated with LED at a wavelength of 735 nm and an energy density of 40 J/cm2. Tumor volume was monitored for 14 days. Data are represented as mean ± SEM (n = 7 per group). Statistical significance was determined using Holm–Sidak multiple comparison test and set at **p < 0.01.

Tumor volume was measured every 2–3 days for 14 days post-PDT to evaluate the tumor growth inhibitory effect of Glc-TFPB-PDT. Glc-TFPB-PDT significantly suppressed tumor growth compared to that in the untreated control group (**p < 0.01). Furthermore, tumor growth inhibition was significantly higher when PDT was performed 24 h after Glc-TFPB administration than when it was performed 2 h after Glc-TFPB administration (**p < 0.01; Fig. 6).

## Discussion

In this study, we evaluated the efficacy of a novel bacteriochlorin derivative, Glc-TFPB, for PDT using both in vitro and in vivo models. Bacteriochlorins are tetrapyrrole compounds with two reduced pyrrole rings in their macrocycle that are characterized by strong absorption in the near-infrared (NIR) region (700–800 + nm), which facilitates deep tissue penetration^30–33^.

Here, Glc-TFPB exhibited a maximum absorption wavelength of 760 nm, with excitation occurring at a longer wavelength than that of TS (660 nm). NIR window, known as the “therapeutic window,” minimizes tissue absorption and scattering, facilitating deep light penetration and enhanced therapeutic effects^34,35^. In animal tissues, light at 630 nm penetrates to less than 5 mm depth, whereas light at 700 nm reaches depths of approximately 8 mm^36–38^. These optical properties highlight the advantages of NIR-absorbing PSs, such as Glc-TFPB, for deep-seated tumor treatment.

Generally, regardless of whether they are chlorins or bacteriochlorins, hydrophobic PSs readily cross cell membranes and tend to localize mainly in the mitochondria^39^. In contrast, hydrophilic PSs are typically internalized via indirect uptake pathways, such as endocytosis, and mainly localized in lysosomes^30,40^. Nevertheless, this tendency is not universal and varies depending on the molecular structure, chemical modifications, and cell type.

Subcellular localization of bacteriochlorin derivatives depends on their chemical structures, with distribution observed in both lysosomes and mitochondria^39,41^. Differences in hydrophobicity and hydrophilicity determine their cellular uptake pathways and intracellular distribution, thereby affecting the therapeutic efficacy of PDT^42^. Some studies have indicated that bacteriochlorin derivatives reach maximal uptake in tumor cells approximately 24 h after administration and exhibit high tumor accumulation even at 72 h^43–45^. Nevertheless, the precise uptake mechanisms of these derivatives remain unclear.

TS localizes primarily to lysosomes, with cellular uptake occurring via ATP-dependent clathrin- and caveolae-mediated endocytosis, accompanied by K-Ras activation^46,47^. Previous studies using other lysosome-targeting PSs have demonstrated that the ROS generated by PDT increase lysosomal membrane permeability, thereby promoting the release of lysosomal enzymes into the cytosol and subsequently activating the apoptosis-related signaling pathways^48–51^. Although this sequence of events has not been directly demonstrated in TS-PDT, its predominant lysosomal localization suggests the involvement of a similar mechanism. Glc-TFPB, which also exhibits predominant lysosomal localization, possibly operates through a comparable mechanism. Notably, Glc-TFPB showed minimal mitochondrial localization, likely due to its increased hydrophilicity and high molecular weight resulting from glycosylation. These physicochemical properties suggest that Glc-TFPB uptake occurs via mechanisms other than passive diffusion, including alterations in membrane fluidity, protein-mediated transport, and specific endocytic pathways.

PDT cytotoxicity is primarily mediated by ROS generation, which damage cellular components and induce cancer cell destruction^52,53^. Therefore, efficient ROS production is crucial for maximizing the efficacy of PDT. Notably, at a low concentration of 5 µM in vitro, TS-PDT induced minimal ROS generation, whereas Glc-TFPB-PDT significantly increased the intracellular ROS levels. The absence of detectable ROS in TS-PDT can be attributed to its higher IC₅₀ value at 24 h post-administration (13.60 ± 1.98 µM) compared with that of Glc-TFPB-PDT (1.10 ± 0.08 µM), indicating lower phototoxic efficiency at 5 µM. This result is consistent with previous reports that bacteriochlorins produce ROS more efficiently than chlorins^24^.

Interestingly, Glc-TFPB-PDT exhibited lower cytotoxicity than TS-PDT at 2 h post-administration but higher cytotoxicity than TS at 24 h post-administration. This result possibly reflects the uptake kinetics of Glc-TFPB, which plateaued at approximately 24 h in both the in vitro and in vivo models. Although definitive reports on the plateau time of TS uptake in vitro are lacking, some studies have reported its relatively rapid uptake^47,54^. In vivo mouse models have shown that TS achieves maximal tumor accumulation approximately 2 h post-administration, consistent with our observation of comparable TS-PDT cytotoxicity between 2 and 24 h.

Glycosylation of bacteriochlorin derivatives improves tumor cell selectivity and uptake efficiency in vitro^51^. Therefore, glycosyl moiety of Glc-TFPB possibly contributes to its enhanced tumor selectivity and accumulation. In our in vivo PDT experiments, the group receiving Glc-TFPB 24 h before treatment exhibited significantly higher tumor growth suppression than the control group and the group treated 2 h after Glc-TFPB administration. This enhanced efficacy was possibly due to the synergistic effects of sufficient tumor accumulation and deep tissue penetration enabled by 760 nm excitation.

Collectively, our findings suggest that Glc-TFPB offers considerable advantages over TS in terms of tumor selectivity, treatment depth, and photophysical properties, positioning it as a promising next-generation PS for PDT.

This study has several limitations. First, a single human colorectal cancer cell line (HCT116) and xenograft mouse model were used for the experiments, which possibly limits the generalizability of our findings to other tumor types or clinical settings. Second, the specific mechanisms underlying Glc-TFPB uptake and intracellular trafficking could not be fully elucidated in this study. Although lysosomal localization was observed, additional studies using inhibitors or gene silencing approaches are necessary to clarify the precise pathways. Finally, potential off-target effects, toxicity in normal tissues, and systemic pharmacokinetics were not comprehensively evaluated in this study. Future studies should address these limitations to facilitate the successful clinical translation of Glc-TFPB-PDT.

## Conclusion

In this study, we demonstrated that the novel glucose-conjugated bacteriochlorin derivative, Glc-TFPB, exhibits superior photophysical and biological properties compared with TS. Glc-TFPB showed strong absorption in the near-infrared region, efficient ROS generation, and lysosomal localization, leading to potent apoptosis induction upon irradiation. Furthermore, in vivo experiments confirmed that Glc-TFPB preferentially accumulates in tumors and that PDT administered 24 h post-injection results in significant tumor growth suppression. These findings highlight Glc-TFPB as a promising next-generation PS that could expand the clinical applicability of PDT, particularly for gastrointestinal and potentially other malignancies.

## Supplementary Information

Supplementary Information 1.

Supplementary Information 2.

## Data Availability

All data generated or analysed during this study are included in this published article and its supplementary information files.
