# Supplementary material for: Antitumor effects of a novel glucose-conjugated bacteriochlorin for photodynamic therapy
Source: Sci Rep. 2025 Nov 25;15:45342. doi: 10.1038/s41598-025-29901-7 (PMC12748749; doi:10.1038/s41598-025-29901-7)
Supplement: Supplementary file 2 — Supplementary Information 2. [file 41598_2025_29901_MOESM2_ESM.pdf]

A

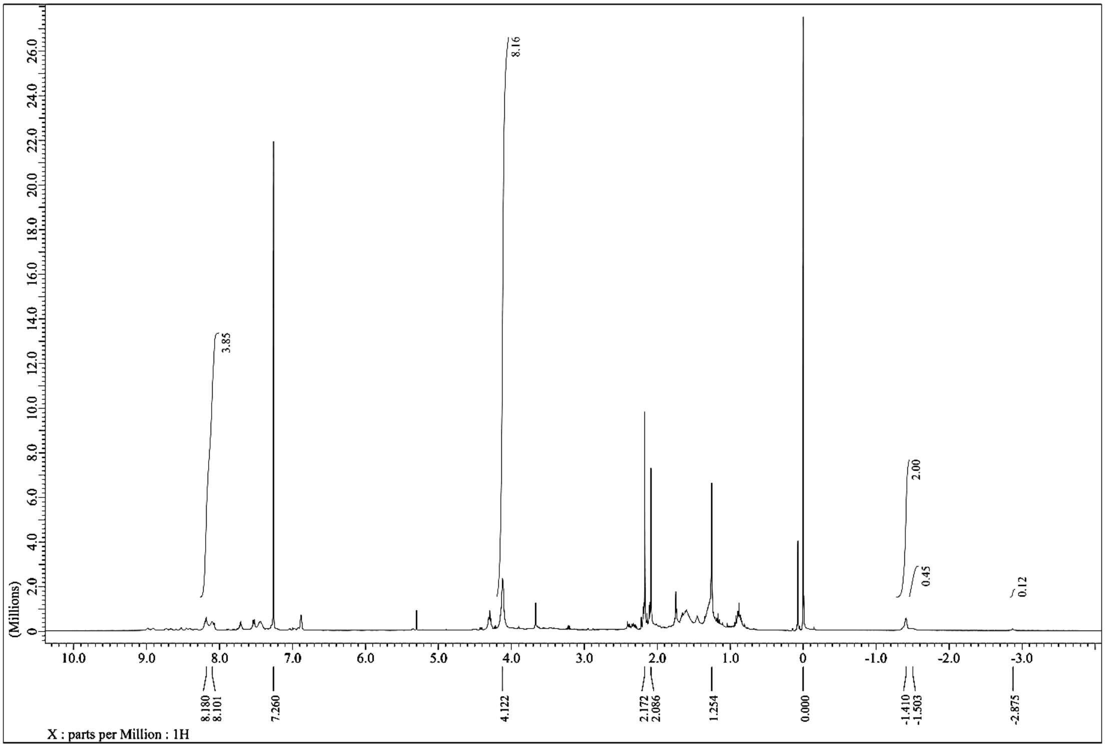

B

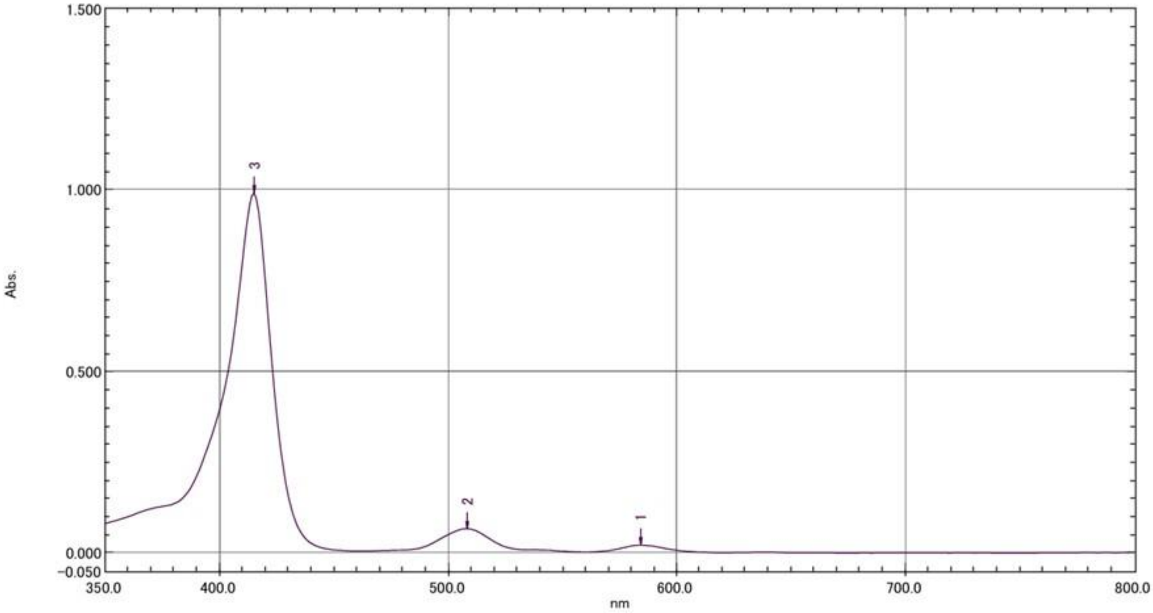

Supplementary Figure 1 | Characterization of Glc-TFPB.
(A) ^{1}H NMR of Glc-TFPB.
(B) UV-Vis. spectrum of starting material (0.5×10^{-5} M in CH_2Cl_2).

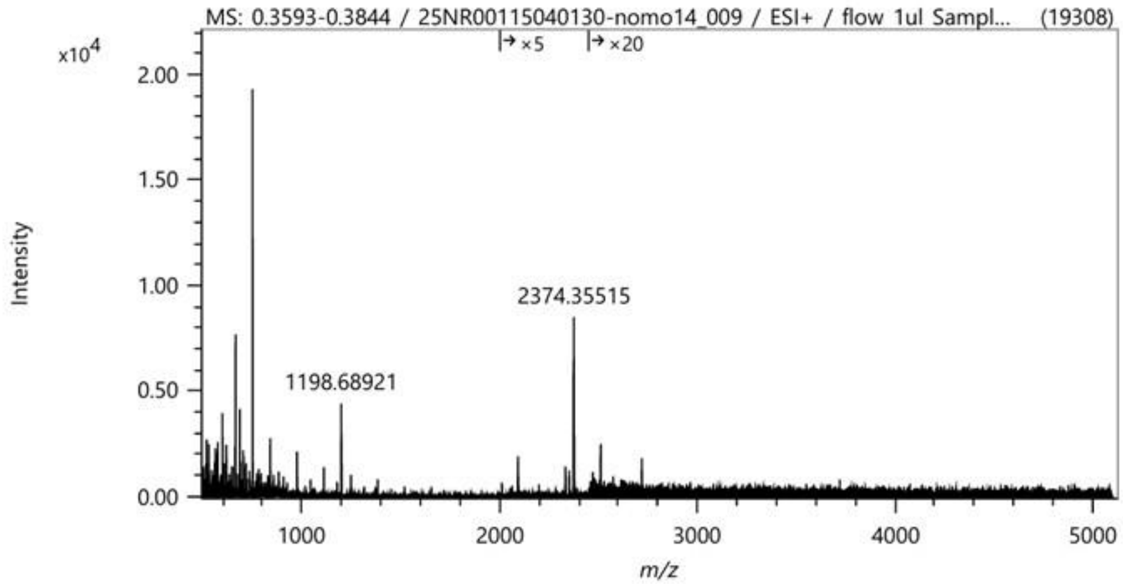

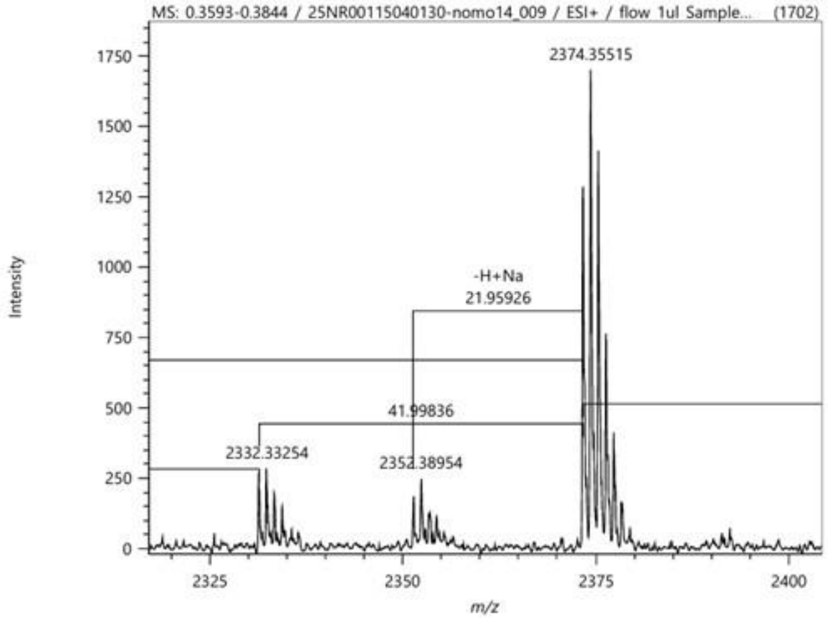

Elemental Composition

Parameters

Tolerance:	±2.00 mDa
Electron:	Odd/Even
Charge:	+1
DBE:	-1.5 - 52.0

Elements Set 1:

Symbol	C	H	O	N	Na	S	F
Min	95	0	0	0	0	4	0
Max	102	90	38	4	1	4	16

Results

Mass	Intensity	Formula	Calculated Mass	Mass Difference [mDa]	Mass Difference [ppm]	DBE
2351.39139	186.00	C100 H90 N4 O37 F15 S4	2351.39217	-0.78	-0.33	50.5
2373.35065	1287.28	C100 H87 N3 O38 F15 Na S4	2373.35031	0.34	0.14	51.0

Supplementary Figure 2 | Mass spectra of starting porphyrin.
ESI-Mass. Calcd for $C_{100}H_{86}F_{16}N_4O_{36}S_4Na$ $[M+Na]^+$: 2373.3547. Found: 2373.3507.

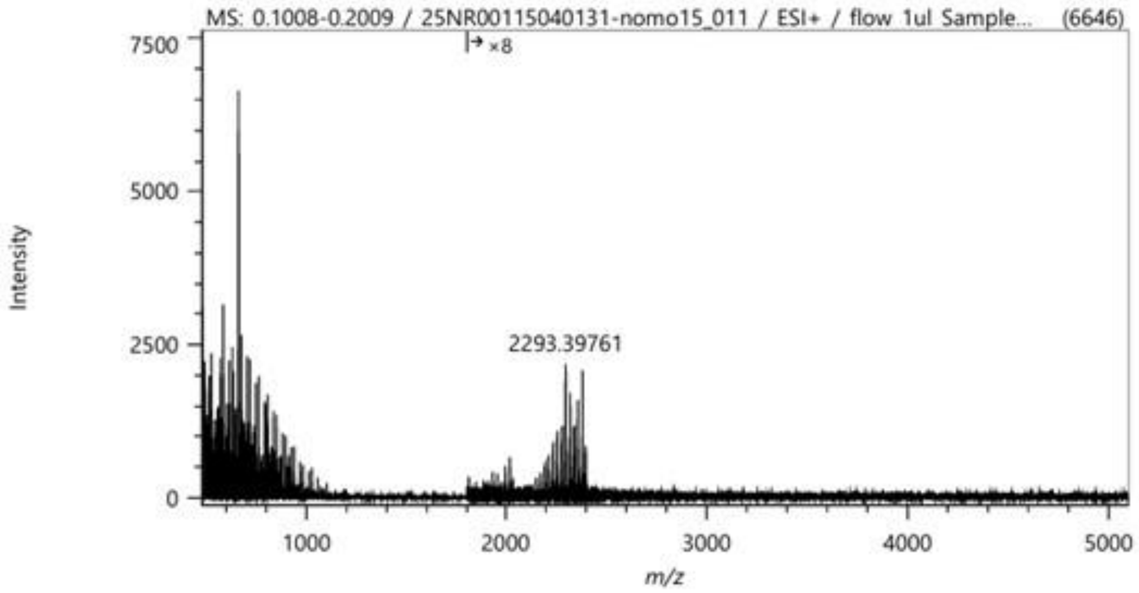

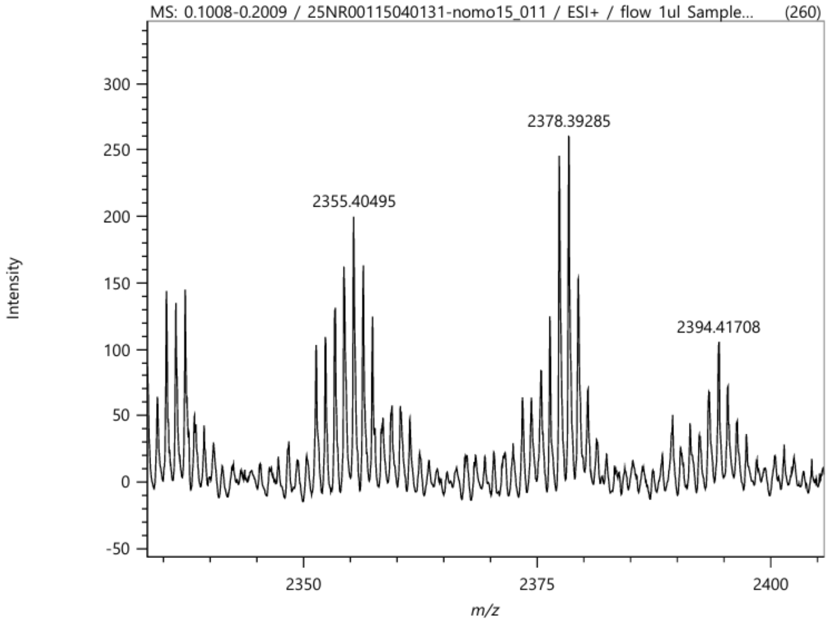

Elemental Composition

Parameters	
Tolerance:	±2.00 mDa
Electron:	Odd/Even
Charge:	+1
DBE:	-1.5 - 55.0

Elements Set 1:

Symbol	C	H	O	N	Na	S	F
Min	95	0	0	0	0	4	0
Max	102	90	38	4	1	4	16

Results

Mass	Intensity	Formula	Calculated Mass	Mass Difference [mDa]	Mass Difference [ppm]	DBE
2377.38386	245.55	C102 H90 N3 O38 F15 S4	2377.38401	-0.16	-0.07	52.0
		C100 H90 N4 O36 F16 Na S4	2377.38543	-1.57	-0.66	49.5

Supplementary Figure 3 | Mass spectra of acetyl-protected bacteriochlorin derivative. ESI-Mass. Calcd for $C_{100}H_{90}F_{16}N_4O_{36}S_4Na$ $[M+Na]^+$: 2377.3860. Found: 2377.3839.
